# Factors influencing the proliferation of keratinocytes in psoriasis

**DOI:** 10.1016/j.isci.2026.114745

**Published:** 2026-01-20

**Authors:** Nannan Liang, Kaiming Zhang

**Affiliations:** 1 Shanxi Key Laboratory of Stem Cell for Immunological Dermatosis, Institute of Dermatology, Peking University First Hospital Taiyuan Hospital, Taiyuan, Shanxi, China

**Keywords:** Health sciences

## Abstract

The pathogenesis of psoriasis is extremely complex, and abnormal proliferation of keratinocytes (KCs) is one of its key pathological features. During the disease process, multiple factors can induce KC proliferation. This article reviews the mechanisms by which inflammatory cytokines, immune microenvironment, microbiome, epigenetics, metabolism, and the autophagy influence KC proliferation, aiming to provide insights for further research into the pathogenesis of psoriasis and the development of precise clinical treatments.

## Graphical abstract

**Figure:**
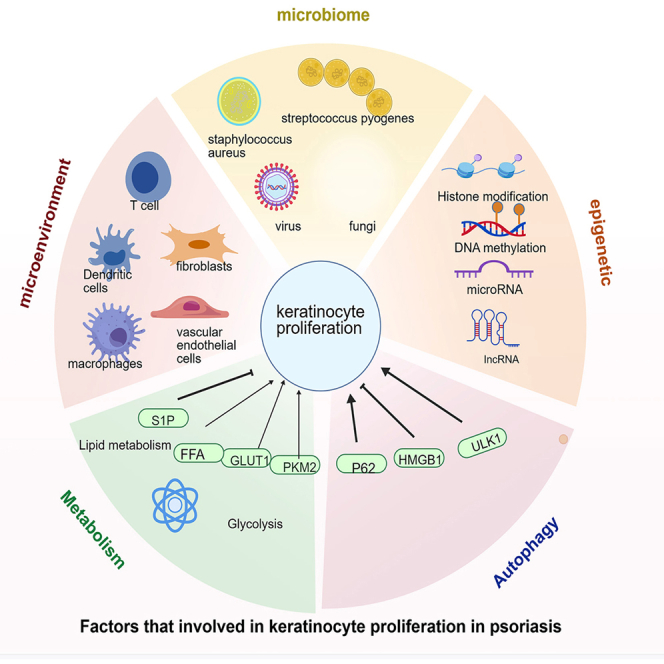

## Introduction

### Psoriasis

Psoriasis is a chronic, immune-mediated, inflammatory, and systemic disease, with a global prevalence of approximately 2%–3%, and its incidence has shown a steadily rising trend in recent years.^1^^,^^2^ From 1990 to 2021, global psoriasis prevalence increased from 23.06 million to 42.98 million (an 86% rise), and incidence grew by 80% from 2.85 million to 5.10 million.^3^ This global rising trend is similar to the rise in specific regions of the world. Both high-socio-demographic index (SDI) regions (including Western Europe, high-income North America, and Andean Latin America) and low-SDI regions (such as East Asia and sub-Saharan Africa) have experienced a steady growth in prevalence, incidence, and disease burden rates from 1990 to 2021.^3^ However, the prevalence of psoriasis varies greatly among regions, ethnic groups, and socioeconomic conditions (Table 1). The prevalence of psoriasis varied from 0.14% (95% uncertainty interval: 0.05%–0.40%) in east Asia to 1.99% (0.64%–6.60%) in Australasia. The prevalence of psoriasis was also high in Western Europe (1.92%, 1.07%–3.46%), Central Europe (1.83%, 0.62%–5.32%), North America (1.50%, 0.63%–3.60%), and high-income southern Latin America (1.10%, 0.36%–2.96%).^4^

**Table 1:** Trends in the prevalence of psoriasis in major global regions by October 2019

Region	Overall Prevalence (%)	95% Uncertainty Interval
Australasia	1.99%	0.64%–6.60%
Western Europe	1.92%	1.07%–3.46%
Central Europe	1.83%	0.62%–5.32%
North America	1.50%	0.63%–3.60%
High-income southern Latin America	1.10%	0.36%–2.96%
East Asia	0.14%	0.05%–0.40%
Global average	2%–3%	

### Risk factors

The etiology of psoriasis is complex and still not fully understood, involving genetic susceptibility, changes in lifestyle, and environmental triggers.

#### Genes

Heritability is the main risk determinant for developing psoriasis. More than 100 genetic polymorphisms related to psoriasis susceptibility or protective properties have been identified.^5^ The HLA-C∗06:02 allele is present in more than 60% of patients and increases the risk for psoriasis.^6^ In addition, genome-wide association studies have identified key non-HLA susceptibility genes, including *IL23A*, *IL12B*, and *IL17RA*, which contribute to the occurrence and development of diseases as well as the response to targeted therapy.^7^

#### Changes in lifestyle

Obesity is a recognized risk factor that contributes to the rising global prevalence of psoriasis.^8^ Studies have also shown that the body mass index (BMI) is positively correlated with psoriasis. Adipose tissues secrete pro-inflammatory adipokines, leading to a low-grade inflammatory state, thereby participating in the pathogenesis of psoriasis.^9^

Smoking and alcohol consumption have been proven to be associated with psoriasis. Smoking is closely linked to pustular skin lesions in psoriasis. Smoking increases the risk of psoriasis, and its impact on the severity of the disease is dose dependent.^10^ Excessive alcohol consumption is associated with an increased activity of psoriasis and a poor treatment response.^11^

Mental stress is regarded as a trigger for psoriasis, and many psoriasis patients and doctors also believe that stress can aggravate the condition. Psychological stress may trigger the onset of psoriasis by disrupting the regulatory mechanism of the hypothalamic-pituitary-adrenal (HPA) axis.^12^

#### Environmental factors

Psoriasis pathogenesis depends on gene-environment interactions, with disease manifestation requiring environmental triggers such as infections, medication exposure, and air pollutants.

One of the most well-known triggers is infection, particularly streptococcal infection, which has been linked to the development of guttate psoriasis, especially in children.^13^ Besides, viral infections may also trigger new psoriasis lesions or aggravate existing conditions.^14^

Some drugs including beta-blockers, lithium agents, antimalarial drugs, and interferons may induce or aggravate psoriasis.^15^

A variety of air pollutants, such as polycyclic aromatic hydrocarbons, volatile organic compounds, oxides, heavy metals and ultraviolet rays, cause damage to the skin by inducing oxidative stress. Pollutants such as air pollution and chemical substances in water can stimulate the skin and immune system, potentially increasing the susceptibility to psoriasis.^16^

### Keratinocyte proliferation in the pathogenesis of psoriasis

The clinical manifestations are diverse, with typical symptoms including scaly erythematous patches or plaques that may be localized or widespread. Based on clinical features, psoriasis is classified into several subtypes—plaque psoriasis (vulgaris), psoriatic arthritis, pustular psoriasis, and erythrodermic psoriasis.^17^ Psoriasis is characterized by a prolonged disease course and a high relapse rate. It can also lead to various complications, including metabolic syndrome, cardiovascular diseases, and psychiatric disorders, significantly impairing patients’ quality of life. The pathological hallmark of psoriasis involves epidermal hyperplasia driven by the excessive proliferation of keratinocytes (KCs). However, the exact pathogenesis remains incompletely understood. Multiple factors including environmental triggers, genetic predisposition, and immune dysregulation, contribute to disease development. Notably, both innate and adaptive immunity play crucial roles in psoriasis pathogenesis, with various immune cells and cytokines being closely associated with its pathophysiological mechanisms.

Keratinocytes are the main component of the epidermis. In addition to being the mechanical protective barrier of the human body, they are the key cells that initiate, maintain, and regulate the skin’s immune response.^18^ Keratinocytes undergo intricately regulated mechanisms during their differentiation and maturation. Even subtle alterations in their microenvironment can lead to dysregulated proliferation and apoptosis, subsequently triggering pathophysiological responses. The keratinocytes in the lesion areas of patients with psoriasis show a state of growth disorder, which is specifically manifested as the thickening of the skin, shortening of the mitotic cycle, shortening of the epidermal passage time, and delay in keratinocyte differentiation at the same time. Later, these changes lead to an increase in the number of proliferating cells in the epidermis.

## Factors that affect keratinocyte proliferation

Researchers have demonstrated that numerous stimulants affect keratinocyte proliferation, including inflammation cytokines, epigenetics, metabolism, immune microenvironment, and inflammatory molecules, among many others. Accordingly, targeting keratinocyte proliferation is considered beneficial in the treatment of psoriasis lesions.

### Extracellular cytokine pathways

#### TNF-α/IL-23/IL-17

In the pathogenesis of psoriasis, immune cells and keratinocytes play a crucial role through communication via cytokines and receptors. Immune cells produce TNF-α, interferon-γ (IFN-γ), interleukin-23 (IL-23)/interleukin-17A (IL-17A), and interleukin-22 (IL-22), which activate keratinocytes, triggering signaling pathways that lead to their excessive proliferation and the production of various active substances. Notably, TNF-α, IL-17A, and IL-23 stand as core pathogenic factors in the disease process, and targeted therapies against these molecules have demonstrated substantial therapeutic efficacy in clinical practice.

IL-23 is a heterodimeric pro-inflammatory cytokine consisting of two subunits, P40 and P19, and it is predominantly expressed by plasmacytoid dendritic cells (pDCs), macrophages, monocytes, T cells, B cells, and keratinocytes. This cytokine exerts biological effects by binding to the IL-23 receptor (IL-23R) on the surface of CD4^+^ T cells. Notably, pro-inflammatory factors such as transforming growth factor β (TGF-β) can upregulate the expression levels of IL-23R and interleukin-1 receptor type 1 (IL-1R1) on CD4^+^ T cells.^19^^,^^20^ Recently, Li et al. demonstrated that keratinocyte-derived IL-23 is sufficient to activate IL-17-producing immune cells, inducing IL-17 secretion, and driving chronic cutaneous inflammation. Furthermore, accumulating evidence indicates that genetic regulation mediated by H3K9 dimethylation modulates IL-23 expression in keratinocytes, which may contribute to the pathogenesis of psoriasis.^21^

The IL-17 family contains six structure-related cytokines: IL-17A to IL-17F.^22^ IL-17A plays the most significant role in the pathogenesis of psoriasis.^23^ After binding to receptors on keratinocytes, IL-17A induces keratinocytes to produce antimicrobial peptides (such as S100A7, LL37, and DEFB4A) to activate innate immunity; produce chemokines (such as CXCL1, CXCL8, and CCL20) to recruit leukocytes such as neutrophils, Th17 cells, myeloid dendritic cells, and macrophages; and express a variety of pro-inflammatory genes (such as IL-1β, IL-6, IL-8, and TNF-α), thereby amplifying the IL-23/IL-17A axis and forming a “feedforward” inflammatory loop. Studies have also found that IL-17A can upregulate galectin-8, which further promotes keratinocyte proliferation by regulating mitosis.^24^ Studies have shown that IL-17A and IL-17F can strongly induce the expression and release of S100A8 and S100A9,^25^ thereby promoting keratinocyte proliferation. In addition, IL-17 can promote keratinocyte stemness and thus promote their proliferation.^26^

TNF-α is another pivotal inflammatory cytokine in the pathogenesis of psoriasis. Produced by keratinocytes, dendritic cells, neutrophils, mast cells, as well as NKT, Th1, Th17, and Th22 cells, TNF-α exerts a dual regulatory effect. On one hand, it significantly suppresses IFN-α secretion by pDCs,^27^ providing a mechanistic explanation for the paradoxical exacerbation or *de novo* development of psoriasis observed during anti-TNF-α therapy.^28^ On the other hand, TNF-α promotes pDC maturation toward conventional dendritic cell phenotypes that produce IL-23.^29^ Furthermore, TNF-α acts synergistically with IL-17A to induce the expression of psoriasis-associated cytokines and keratinocyte-specific genes, thereby modulating keratinocyte biological functions.^30^ The TNF-like weak apoptosis inducer TWEAK (TWEAK), a member of the TNF superfamily, also serves as a key pathogenic factor. Genetic deficiency of TWEAK or its receptor has been shown to attenuate psoriatic dermatitis. Keratinocytes are the core of its action. Blocking TWEAK has similar effects to blocking TNF-α and IL-17A and may become an alternative treatment strategy.^31^

IL-22, another major downstream cytokine of the IL-23 pathway, is predominantly secreted by CD4^+^ T cells and type 3 innate lymphoid cells (ILC3s). Its cognate receptor, IL-22 receptor (IL-22R), is expressed on non-hematopoietic cells, including keratinocytes, epithelial cells, and hepatocytes.^32^ IL-22 exerts pathogenic effects in psoriasis by suppressing the terminal differentiation of keratinocytes and inducing the production of antimicrobial peptides and pro-inflammatory chemokines.^33^ Notably, genetic deficiency of its natural antagonist, IL-22 binding protein (IL-22BP), exacerbates disease severity.^34^

#### IL-36

The interleukin-36 (IL-36) family is a member of the IL-1 superfamily. Johnston et al. confirmed that IL-36α, IL-36β, IL-36γ, and IL-36Ra are upregulated in psoriasis lesions. The IL36RN gene mutation is closely related to pustular psoriasis.^35^ Studies have found that keratinocytes are the main source of IL-36, which acts on target cells through the IL-36R receptor. Activated IL-36 can promote the production of various cellular chemokines and antimicrobial peptides, forming a self-amplifying inflammatory cycle. Mature IL-36 can not only induce T cell proliferation and Th1/Th17 cell differentiation^36^^,^^37^^,^^38^ but also promote the phenotypic and functional maturation of dendritic cells. Therefore, drugs targeting IL-36 or key molecules in its related signaling pathway represent a new therapeutic direction for pustular psoriasis.

### Intracellular signaling pathways of transmission

**Figure 1:**
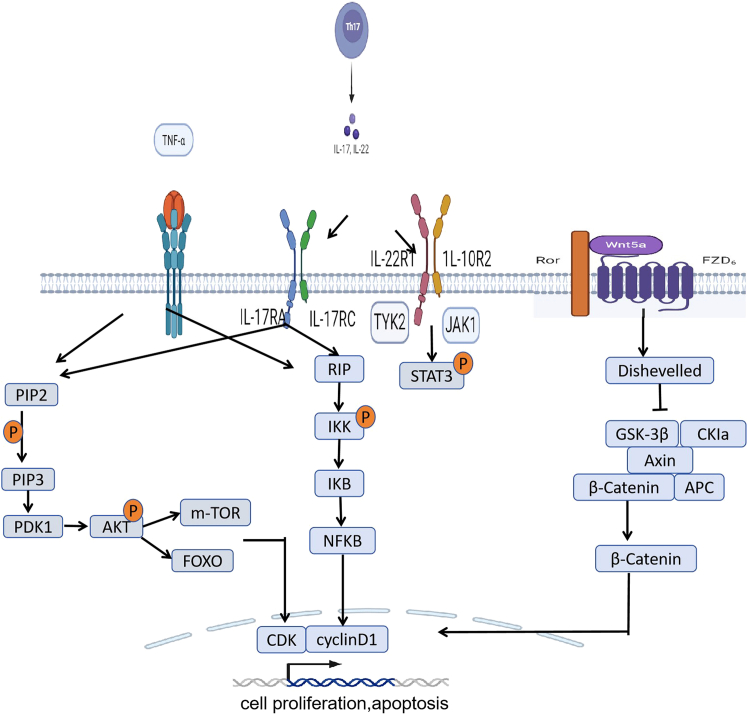
The signaling pathway involved in keratinocyte proliferation in psoriasis Proinflammatory cytokines such as IL-17A and TNF-α bind to their receptors to activate the PI3K/AKT and NF-κB signaling pathways, affecting CDK and cyclin D1 expression to induce cell proliferation. The IL-22 receptor 1/IL-10 receptor 2 (IL-22R1/IL-10R2) heterodimer activates STAT3 by phosphorylating tyrosine kinase 2 (TYK2) and JAK1 and induces proliferation. Activation of Wnt5a signaling increases the phosphorylation level of GSK3β within this complex, reducing its inhibitory effect on β-catenin. This leads to the translocation of β-catenin into the nucleus, where it regulates downstream targets such as cyclin D1 and c-Myc, thereby modulating keratinocyte proliferation.

Cellular signal transduction pathways play a significant role in the etiology, pathological mechanism, progression, and prognosis of psoriasis, and thus have become a research hotspot in the field related to psoriasis at present. Among them, the PI3K/AKT signaling pathway, JAK/STAT signaling pathway, and Wnt/β-catenin signaling pathway play significant roles in the keratinocyte proliferation in psoriasis (Figure 1).

#### PI3k/AKT pathway

The phosphatidylinositol 3-kinase (PI3K)/ protein kinase B (AKT) signaling pathway is involved in multiple cellular processes such as cell growth, proliferation, angiogenesis, energy metabolism, and tumor cell invasion. Studies have shown that, compared to normal skin, the expression levels of p-PI3K and p-AKT are significantly upregulated in psoriasis lesions.^39^ Upon activation by receptor tyrosine kinases (RTKs), PI3K generates phosphatidylinositol 3,4,5-trisphosphate (PIP3), a lipid second messenger that recruits AKT to the plasma membrane. At the membrane, AKT undergoes complete activation through sequential phosphorylation—Thr308 phosphorylation mediated by 3-phosphoinositide-dependent protein kinase 1 (PDK1), and Ser473 phosphorylation catalyzed by mammalian target of rapamycin complex 2 (mTORC2). The activated AKT then translocates to orchestrate psoriasis pathogenesis by modulating downstream signaling pathways.^40^ The FOXO transcription factor is negatively regulated by phosphorylated AKT, and activated FOXO can inhibit cell proliferation.^41^ Studies have shown that FOXO in psoriasis lesions is mainly localized in the cytoplasm, whereas it is present in the nucleus in normal skin cells. Studies have confirmed that the transcriptional activity of FOXO can be regulated by nuclear translocation.^42^ The excessive activation of p-AKT may change the localization of FOXO, causing it to translocate from the nucleus to the cytoplasm, resulting in a weakened inhibitory effect on cell proliferation and thereby causing excessive proliferation of keratinocytes.^43^

The downstream of the PI3K/AKT signaling pathway is not only FOXO but also mTOR (the target protein of mammalian rapamycin). It can also regulate cell proliferation and apoptosis by phosphorylating downstream proteins such as mTOR. On one hand, activation of the PI3K/AKT signaling pathway stimulates downstream mTORC1, which phosphorylates key translation initiation proteins, S6 kinase 1 (S6K1) and eukaryotic initiation factor 4E-binding protein 1 (4E-BP1), thereby enhancing the rate of protein biosynthesis. This process facilitates the G1 to S phase transition in the cell cycle and accelerates keratinocyte proliferation.^44^ On the other hand, the PI3K/AKT pathway suppresses autophagy through mTORC1 activation. As autophagy is essential for the terminal differentiation of keratinocytes into corneocytes, hyperactive mTORC1 impairs nuclear degradation and disrupts normal differentiation, leading to both aberrant proliferation and impaired differentiation of keratinocytes.^45^ Mitra et al. found that IL-22-induced cell proliferation is regulated by the PI3K/AKT/mTOR signaling cascade. The PI3K/AKT/mTOR pathway can also regulate the Th1/Th2/Th17 imbalance in psoriasis.^46^ Besides, mTOR participates in the secretion of pro-inflammatory molecules, such as CXCL8, IL-6, and VEGF, by keratinocytes.^47^

#### JAK/STAT pathway

The JAK/STAT signaling pathway is involved in a range of autoimmune diseases, including rheumatoid arthritis, inflammatory bowel disease, and psoriasis. Various cytokines stimulate different isoforms of JAK protein kinases, namely JAK1, JAK2, and JAK3, which then activate STAT transcription factors to target a series of genes. Various cytokines bind to transmembrane cytokine receptors, inducing receptor dimerization. This process activates JAK kinases and exposes the binding sites for STAT proteins, facilitating the recruitment of STAT proteins to the receptor through SH2 domains. Subsequently, the activated STAT proteins form phosphorylated STAT dimers. These dimers then translocate to the nucleus and bind to the promoter regions of specific target genes.^48^ IL-22 increases the phosphorylation of JAK1 and Tyk2, resulting in an increase in STAT3 phosphorylation, thus leading to keratinocyte proliferation and migration in psoriasis.^49^ IFN-γ regulates various cellular functions of keratinocytes in psoriasis through IFN-γ receptor (IFNGR) and JAK/STAT. IFN-γ leads to an increase in JAK1 and JAK2 phosphorylation, resulting in an increase in STAT1 phosphorylation.^50^ STAT3 plays a regulatory role in Th17 cell differentiation and keratinocyte proliferation via JAK1/JAK2 or JAK1/TYK2 signaling pathways triggered by IL-6.^51^ A transgenic mouse model with keratinocyte-specific overexpression of STAT3 spontaneously developed psoriasis-like lesions.^52^ Furthermore, specific ablation of STAT3 in keratinocytes, but not in T cells, attenuated IMQ-induced psoriasis-like dermatitis.^53^

#### Wnt5a/β-catenin

Wnt5a is one of the activators of the Wnt signaling pathway and can regulate cell proliferation, polarity, migration, differentiation and inflammation.^54^ Compared to normal skin, Wnt5a and its 7-transmembrane receptor Frizzled2/5 are highly expressed in psoriasis lesions. Dou et al. (2017) demonstrated, via RNA sequencing and comprehensive analysis of psoriasis skin samples, that *Wnt5a* is a key pathogenic gene in psoriasis and holds potential as a biomarker for the diagnosis and therapeutic monitoring of the disease.^55^ Treatment of keratinocytes with recombinant human Wnt5a can produce proliferation-promoting effects *in vitro* and increase the secretion of IL-23, IL-12, and TNF-α, promoting the development and progression of psoriasis.^56^ Studies indicate that Wnt5a can influence keratinocyte proliferation and apoptosis through either the canonical Wnt/β-catenin pathway or the non-canonical PKC/Ca^2+^-dependent protein kinase II pathway. In the cytoplasm, β-catenin remains inactive under the regulation of the GSK3β/Axin/APC protein complex.^57^

Activation of Wnt5a signaling increases the phosphorylation level of GSK3β within this complex, reducing its inhibitory effect on β-catenin. This leads to the translocation of β-catenin to the nucleus, where it regulates downstream targets such as cyclin D1 and c-Myc, thereby modulating keratinocyte proliferation. Knockdown of Wnt5a downregulates proliferation markers like PCNA and Ki-67. Additionally, IL-36γ in psoriasis lesions inhibits keratinocyte differentiation and triggers inflammatory responses via the Wnt/β-catenin signaling pathway.^58^

### Immune microenvironment and keratinocyte proliferation

**Figure 2:**
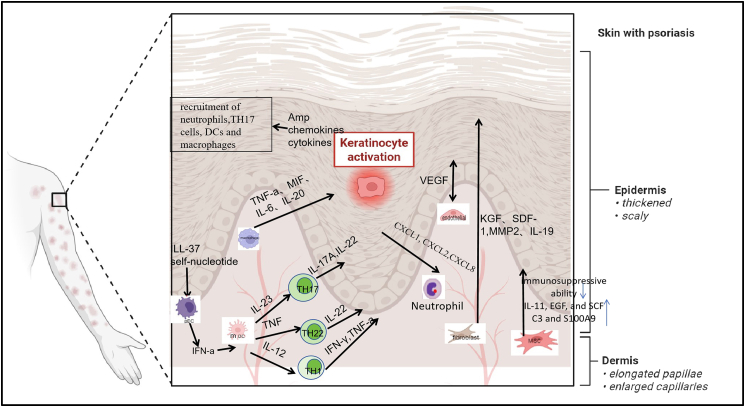
Interactions between keratinocytes and immune cells in psoriasis lesions The pathogenesis of psoriasis involves multiple cellular interactions of the innate and adaptive immune systems, forming a self-amplifying inflammatory circuit. LL-37 complexes activate pDCs to produce IFNs, which stimulate dendritic cells to release TNF, IL-12, and IL-23. These cytokines drive naive T cell differentiation into Th1, Th17, and Th22 cells to induces keratinocyte proliferation and chemokine production, thus perpetuating the inflammatory loop. Macrophages may induce excessive keratinocyte proliferation and abnormal differentiation by secreting cytokines such as TNF-α, MIF, IL-6, and IL-20. Fibroblasts significant upregulate the expression of pro-inflammatory, adipogenic, and chemokine genes such as *IL6*, *CXCL8*, *CXCL1*, *CXCL2*, *KGF*, *SDF-1*, and cathepsin S to promote keratinocyte proliferation. Simultaneously, activated endothelial cells recruit neutrophils and Th17 cells to the skin by expressing adhesion molecules (ICAM-1 and VCAM-1) and secreting chemokines (CXCL1, CXCL8, and CXCL10) to drive keratinocyte hyperproliferation. Ultimately, these cells form a positive feedback loop through the IL-23/IL-17 axis, leading to the typical pathological features of epidermal proliferation, vasodilation, and chronic inflammation. pDCs, plasmacytoid dendritic cells.

Psoriasis is a complex disease, involving a dynamic interplay between immune cells, keratinocytes, and various other skin-resident cells, such as endothelial and fibroblast cells.^59^ Single-cell technology has provided a deeper understanding of the pathogenesis of psoriasis. The technology has further confirmed that keratinocytes exist in multiple states including inflammatory differentiation, proliferative, and undifferentiated phenotypes. Notably, basal layer cells acquire abnormal proliferative capacity through upregulation of keratins such as KRT6/KRT16.^60^ Cheng et al. analyzed a total of 92,889 epidermal cells from three anatomical sites (adult scalp skin, adult trunk skin, and neonatal circumcision) and identified eight clusters related to keratinocytes, indicating that keratinocytes exhibit unique gene expression patterns in different anatomical sites.^61^ A new subpopulation of pathogenic IL-17A^+^IFN-γ^+^ Th17 cells was discovered in psoriasis lesions through single-cell transcriptome sequencing,^62^ revealing phenotypic and functional heterogeneity among CD8^+^ T cells. The CXCL13^+^ Tc17 subset was identified as a lesion-specific population whose abundance positively correlates with disease severity.^63^ Furthermore, tissue-resident memory (TRM) T cells, endowed with intrinsic memory of psoriasis lesions, have been identified as key contributors to disease recurrence.^64^ The immune microenvironment plays a vital role in keratinocyte proliferation (Figure 2).

#### Interaction between mesenchymal stem cells and keratinocytes

Mesenchymal stem cells (MSCs) are multipotent progenitor cells that can be derived from various tissues such as bone marrow, placenta, umbilical cord, and adipose tissue, with the ability of self-renewal, multilineage differentiation, and immunomodulation. Studies have also shown that MSCs from psoriasis lesion play a role in the proliferation of keratinocytes. Chang et al.^65^ discovered that psoriasis Dermal Mesenchymal Stem Cells (DMSCs) were more potent than normal DMSCs (n-DMSCs) in stimulating keratinocyte proliferation and secreting proinflammatory cytokines but weaker in promoting apoptosis. Liang et al. discovered that p-DMSCs promote HaCaT cell proliferation, differentiation, and migration by activating the PI3K/AKT signaling pathway. Compared with n-DMSCs, the p-DMSCs showed increased secretion of IL-11, EGF, and SCF.^66^ Moreover, studies have demonstrated that the co-culture of normal human epidermal keratinocytes with p-DMSCs stimulated keratinocyte proliferation and glycolysis but reduced keratinocyte junctions.^67^ The expression level of complement C3 and S100A9, important molecules in the complement pathway, was increased in the psoriasis lesions, with an increase in keratinocyte proliferation and inflammatory response of psoriasis. Co-culture of Normal Human Epidermal Keratinocytes (NHEKs) with p-DMSCs upregulated the expression of C3 and S100A9, providing further evidence for the pathogenesis of psoriasis induced by DMSCs.^68^

#### ***Interaction between (***dendritic cell-T cell interaction and keratinocyte proliferation

In the pathological progression of psoriasis, the aberrant interactions and signal amplification among dendritic cells (DCs), Th17 cells, Th1 cells, neutrophils, and keratinocytes (KCs) constitute a core regulatory network that drives keratinocyte hyperproliferation and chronic inflammation.

As the “initiators” of inflammation, dendritic cells are significantly enriched in psoriasis lesions. Their activation begins with endogenous signals triggered by cells damaged by external stimuli (microbial infection or mechanical damage); these cells release self-nucleic acids that form complexes with the antimicrobial peptide LL-37, activating dendritic cells via the TLR7/TLR9 pathway.^69^ The release of IFN-α causes the maturation of resident dermal dendritic cells and the differentiation of monocytes into inflammatory dendritic cells (iDCs). iDCs produce interleukins such as IL-23, IL-12, and TNF-α, which strongly activate the differentiation of naive T cells into Th1, Th17, and Th22 to induce the proliferation and abnormal differentiation of keratinocytes.^70^ Additionally, two novel dendritic cell subsets were characterized in the dermis: a cDC2 subpopulation highly expressing IL23A, IL1B, and CXCL8, and a mature regulatory dendritic cell subset (mregDC), uniquely found in psoriasis patients, that secretes high levels of IL-15; both subsets serve as critical drivers of inflammation initiation.^71^^,^^72^ Jiang et al. found that dendritic cell-secreted LGALS9 can be received by CD44^+^ dermal fibroblasts, leading to increased ECM expression that creates a stiffer dermal environment and ultimately enhances keratinocyte proliferation in psoriasis skin.^73^

CD4^+^ T cells play a central role in the pathogenesis of autoimmune diseases, and their excessive activation significantly contributes to the progression of psoriasis. Among them, Th17 and Th1 cells, upon polarization and differentiation, become the primary pathogenic cells in psoriasis.^74^ Activated Th17 cells release large amounts of pro-inflammatory cytokines (IL-17A and IL-22), thereby triggering an inflammatory cascade. Specifically, IL-17A activates keratinocytes, induces neutrophil recruitment, and promotes the secretion of chemokines and pro-inflammatory cytokines.^75^ IL-22 leads to excessive proliferation and abnormal differentiation of keratinocytes, ultimately resulting in epidermal hyperplasia and the formation of characteristic scaly plaques in psoriasis.^76^^,^^77^^,^^78^ IL-23 amplifies cellular responses, further inducing keratinocyte proliferation and other hallmark features of psoriasis.^79^

Th22 cells are a recently identified CD4^+^ T cell subset enriched in human skin. Their primary secretory cytokine, IL-22, promotes epithelial innate immune responses. IL-22 can directly act on keratinocytes, promoting their dryness and excessive proliferation.^80^ Zheng et al. found that IL-22 induces psoriasis-like dermatitis and epidermal spinous hypertrophy by activating the STAT3-mediated IL-23 signaling pathway.^81^

As a major source of TNF, Th1 cells enhance the activity of mitogen-activated protein kinase (MAPK) and NF-κB signaling pathways in IL-1-activated keratinocytes, thereby promoting keratinocyte proliferation.^82^ Furthermore, IFN-γ secreted by Th1 cells promotes the expression of CXCL9, CXCL10, and CXCL11 in keratinocytes via the JAK/STAT signaling pathway, thereby recruiting more T cells to the skin tissue.^83^

Besides, the CXCL13^+^ Tc17 subset was identified as a lesion-specific population whose abundance positively correlates with disease severity.^84^ In psoriasis lesions, TRM cells, particularly CD8^+^ CD103^+^ TRM cells, serve as a potent and persistent source of IL-17A, while CD4^+^ TRM cells primarily produce IL-22. These cytokines directly act on keratinocytes, driving their hyperproliferation and inhibiting normal differentiation.^85^ Furthermore, TRM T cells, endowed with intrinsic memory of psoriasis lesions, have been identified as key contributors to disease recurrence.

In addition to IL-17 derived from T cells, neutrophils are important producers of IL-17A, which can activate keratinocytes to produce chemokines and antimicrobial peptides.^86^ Keratinocytes-derived CXCL1, CXCL2, and CXCL8, induced by IL-17/IL-36, efficiently recruit neutrophils to the lesion area. Neutrophil-released neutrophil extracellular traps (NETs) not only capture pathogens but also activate dendritic cells and Th17 cells, amplifying the immune response.^87^ Neutrophil-secreted elastase and matrix metalloproteinases degrade the extracellular matrix, compromising skin barrier integrity and creating a microenvironment conducive to keratinocyte hyperproliferation.^88^

#### Interaction between macrophages and keratinocyte proliferation

Research has found that macrophages are closely related to psoriasis. Macrophages are involved in the inflammatory process. Activated macrophages release inflammatory factors, chemokines, and antimicrobial peptides^89^; simultaneously, macrophages promote the proliferation and differentiation of keratinocytes, leading to epidermal hyperplasia and parakeratosis.^90^ The proportion of M1/M2 macrophages in the tissues of mouse psoriasis models was found to be relatively high. The study found that CD68^+^iNOS^+^ M1 macrophages were increased and CD68^+^CD163^+^M2 macrophages were decreased in human psoriasis lesional skin compared with skin samples from normal individuals.^91^ Activated macrophages (especially M1 type) secrete a large amount of pro-inflammatory cytokines (TNF-α, MIF, IL-6, and IL-20), which directly act on the surface receptors of keratinocytes and initiate proliferation-related signaling pathways. Studies by Sa et al.^92^^,^^93^ found that macrophage- derived IL-20 is elevated in psoriasis lesions. IL-20 can induce hyperproliferation and aberrant differentiation of keratinocytes both *in vivo* and *in vitro*. By binding to the receptors IL-20R1 and IL-20R2 on keratinocytes, IL-20 promotes epidermal thickening and other psoriasis features, such as STAT3 activation, overexpression of CK16 and S100 protein family members (including S100A7), and upregulation of proteases involved in skin migration and remodeling, ultimately leading to excessive keratinocyte proliferation. Additionally, Rich and Kupper^94^ found that monocytes, the precursors of macrophages, are also a major source of IL-20. Upon lipopolysaccharide (LPS) stimulation, monocytes rapidly increased IL-20 secretion within 2 h, followed by a gradual decline. Gesser et al.^95^ demonstrated elevated levels of macrophage migration inhibitory factor (MIF) in lesional skin of patients with plaque psoriasis. MIF can activate the mitogen and stress-activated kinase (MSK1) pathway and the p90 ribosomal S6 kinase (RSK1) pathway, modulating transcription factors involved in keratinocyte proliferation and thereby contributing to excessive keratinocyte growth in psoriasis lesions. Macrophages and their monocyte precursors are among the key sources of MIF. Thus, in psoriasis, macrophages may promote keratinocyte hyperproliferation through MIF secretion.^96^

Macrophages present pathogen antigens or autoantigens to T cells, inducing the differentiation of Th17 cells and the secretion of IL-17 and IL-22. These cytokines act in synergy with the cytokines secreted by macrophages.^97^ The pro-angiogenic factors (such as VEGF) secreted by macrophages can induce angiogenesis in the lesion area, increase the supply of nutrients and oxygen, and provide microenvironmental support for the abnormal proliferation of keratinocytes.^98^

Besides, macrophages recruited to the injured skin are subsequently induced by CCL2 to produce epidermal growth factor, which, in turn, promotes keratinocyte proliferation and initiates a positive feedback loop.^99^

#### Interaction between vascular endothelial cells and keratinocyte proliferation

In the pathogenesis of psoriasis, IL-17A and TNF-α jointly stimulate skin keratinocytes to release VEGFA, leading to pathological angiogenesis.^100^ IL-36γ was strongly induced by IL-17A and, together with IL-17A, efficiently activated human dermal microvascular endothelial cells.^101^ Activated endothelial cells recruit neutrophils and Th17 cells to the skin by expressing adhesion molecules (ICAM-1 and VCAM-1) and secreting chemokines (CXCL1, CXCL8, and CXCL10). This process fosters a highly inflammatory milieu that potently drives keratinocyte hyperproliferation.^102^ It has been found that insulin-like growth factor binding protein 7 (IGFBP7hi) endothelial cells can degrade the glycocalyx barrier, which enables T cells to infiltrate and leads to the inflammation visible in psoriasis, thus promoting keratinocyte proliferation.^103^

#### Interaction between keratinocyte proliferation and fibroblasts

Dermal fibroblasts, as important supporting cells of epidermal keratinocytes, can regulate the morphological and functional characteristics of keratinocytes.^104^ Fibroblasts and their subtypes, as pro-inflammatory signals, proliferative mediators, and immunomodulatory messengers, play a key role in the pathogenesis of psoriasis. Psoriasis fibroblasts exhibit a unique gene expression profile, characterized by significant upregulation of pro-inflammatory, adipogenic, and chemokine genes such as IL6, CXCL8, CXCL1, and CXCL2.^105^ We focused on the effect of fibroblasts on the proliferation of keratinocytes in psoriasis. Dermal fibroblasts are the main sources of KGF and EDA^+^FN, both of which can promote the abnormal proliferation of keratinocytes.^106^ When psoriasis fibroblasts were treated with IL-17A and TNF-α, they produced higher levels of IL-19, IL-24, and EREG, and subsequently, promoted the proliferation of keratinocytes through the paracrine signaling pathway.^107^^,^^108^ Stromal cell-derived factor-1 (SDF-1) is upregulated in psoriasis, and fibroblasts are the main source of SDF-1 derived from stromal cells. Functionally, SDF-1 activates the ERK pathway to stimulate the proliferation of epidermal keratinocytes.^109^ In addition, psoriasis fibroblasts exhibit increased oxidative stress and elevated levels of ROS, NO, and mitochondrial superoxides, forming an oxidative microenvironment that activates keratinocytes and accelerates their cell cycle process.^110^^,^^111^ The level of S100A8/A9 in psoriasis significantly increased in dermal fibroblasts, indicating that fibroblasts have a significant ability to secrete pro-inflammatory factors and thus promote the proliferation of keratinocytes in psoriasis. Further research has found that SFRP2^+^ fibroblasts secrete proteases, such as cathepsin S, which can activate IL-36γ,^112^^,^^113^ further amplifying the inflammatory response. Fibroblasts with a high expression of matrix metalloproteinase 2 (MMP2), which mediates structural changes in skin tissue and regulates inflammatory responses, can upregulate the expression of CD103. Through the CD100-PLXNB2 signaling axis, these fibroblasts further enhance the residency of CD8^+^ T cells, thereby playing a critical role in the amplification of inflammation and recurrence of psoriasis.^114^ Fibroblast growth factor 7 (FGF7), secreted by fibroblasts, not only exerts a strong promoting effect on the proliferation and migration of keratinocytes but also can induce the expression of interleukin-19 (IL-19). Subsequently, IL-19 secreted by keratinocytes further stimulates fibroblasts to secrete a greater amount of FGF7, thereby forming a positive feedback regulatory loop.^115^

### Epigenetics regulate keratinocyte proliferation

Epigenetic modifications refer to heritable changes in gene function that occur without alterations in the DNA sequence and include DNA methylation, histone modifications, and non-coding RNAs.^116^

#### DNA methylation and keratinocyte proliferation

DNA methylation is one of the earliest identified mechanisms of epigenetic modification in vertebrates. DNA methylation involves the addition of a methyl group to the C-5 in the cytosine residue, which commonly occurs in the context of cytosine-guanine dinucleotides (CpG) in the DNA strand. Multiple studies have revealed differentially methylated gene loci in the lesional skin tissues of psoriasis patients, primarily involving biological processes such as cell proliferation, apoptosis, cell cycle regulation, and immune system modulation.^117^ Genes exhibiting hypermethylation include *PDCD5*, *TIMP2*, *SELENBP1*, *CARD14*, *KAZN*, *ECE1*, *MAN1C1*, *DLGAP4*, and *SFRP4*, while hypomethylated genes include *S100A9*, *PTPN22*, *CYP2S1*, *EIF2C2*, *SHP-1*, and *HLA-DRB1*. The methylation levels of these genes are inversely correlated with their expression.^118^^,^^119^ Sheng et al. found that *CYP2S1* is hypomethylated in the skin tissue of psoriasis. *CYP2S1* may inhibit the proliferation of keratinocytes and regulate the immune response through IL-8, IL-33, IL-36, CXCL-10, and CCL20, thereby participating in the occurrence and development of psoriasis.^120^ Studies suggest that the most likely reason for the increased expression of B cell receptor-associated protein 31 (BCAP31) in psoriasis cells is the demethylation of the BCAP31 promoter, which leads to excessive proliferation of keratinocytes and incomplete keratinization.^121^ The S100A family plays a significant regulatory role in keratinocyte proliferation and inflammatory responses.^122^^,^^123^ Abnormal methylation of the promoter of Wnt inhibitor-1 (WIF1), a molecule that inhibits the Wnt signaling pathway to regulate cell proliferation and differentiation, was reported to cause a decrease in its expression, triggering keratinocyte proliferation and IL-8 production.^124^

#### Histone modifications and keratinocyte proliferation

Histone modifications are crucial epigenetic regulatory mechanisms that influence chromatin structure, thereby modulating gene transcription. Several biochemical modifications of histones have been identified, including acetylation, methylation, ubiquitination, and sumoylation.

Studies have shown that the reduction of H3K9D methylation in the altered keratinocytes of psoriasis is associated with increased expression of IL-23. H3K27 hyperacetylation has been reported to lead to RPL22 upregulation, and this overexpression is associated with enhanced cyclin D1 levels, driving keratinocyte proliferation.^125^ The histone modification H3K27me3 is overexpressed in the epidermis of psoriasis, and suppression of H3K27me3 levels was shown to reduce keratinocyte proliferation, contributing to the improvement of the phenotype in a mouse model of psoriasis.^126^ Studies have shown that histone deacetylases (HDACs) are upregulated in damaged skin and may promote endothelial cell proliferation and keratinocyte survival.^127^ The histone deacetylase SIRT1, which normally suppresses proliferation and promotes keratinocyte differentiation, is downregulated in psoriasis. This deficiency may contribute to the uncontrolled keratinocyte growth observed in psoriasis lesions.^128^^,^^129^ Xia et al. found that increased histone H3 acetylation of the IL17a promoter could promote Th17 and γδ T17 cell differentiation, which contributed to the immune imbalance and development of psoriasis.^130^

Lysine crotonylation of proteins is a newly identified modification that impacts diverse biological processes, and its dysregulation has been implicated in autoimmune diseases. S100A7 plays a pivotal role in the pathogenesis of psoriasis. Liang et al. discovered that crotonylation deficiency of S100A7 K49 promotes psoriasis keratinocyte proliferation through enhanced interaction with RAGE.^131^

Lysine 2-hydroxyisobutylylation (Khib) forms a unique chemical structure, directly mediating environmental effects on the epigenome and other biological processes. Khib has been found in proteins associated with cell proliferation, differentiation, tricarboxylic acid cycle, and glycolysis. ErbB3 binding protein 1 (Ebp1) is encoded by human *PA2G4* gene, which has been highly conserved in the whole evolution process. Li et al. demonstrated that the downregulation of Ebp1Khib210 promotes keratinocyte proliferation through the induction of TIF-IA-mediated rRNA synthesis.^132^

#### MiRNAs and keratinocyte proliferation

MicroRNAs (miRNAs) are a class of small, non-protein-coding single-stranded RNA molecules. To date, more than 250 miRNAs have been found to be dysregulated in psoriasis.^133^ They participate in the regulation of target genes involved in keratinocyte proliferation and differentiation, production of cytokines and chemokines, and immune-inflammatory response-related signaling pathways, thereby playing a crucial regulatory role in the pathogenesis and development of psoriasis (Table 2).

**Table 2:** Main microRNAs altered in psoriasis

name	level	Target	Function	Reference
miR-203	upregulated	TNF-α; IL-24; SOCS-3	promotes keratinocyte proliferation	Xu et al.^134^
miR-17-92	upregulated	CDKN2B; SOCS1	promotes keratinocyte proliferation	Zhang et al.^135^
miR-223	upregulated	PTEN	increases keratinocyte proliferation	Wang et al.^136^
miR-146a	upregulated	CARD10, FERMT1, IRAK1, and TRAF6	diminishes keratinocyte proliferation	Xia et al.^137^
miR-155	upregulated	PTEN	inhibits keratinocyte proliferation and promotes apoptosis	Yao et al.^138^
miR-221/miR-222	upregulated	TIMP3	increases keratinocyte proliferation	Zibert et al.^139^
miR-136	upregulated	PPP2R2A	TGF-β1-induced keratinocyte proliferation arrest	Zhang et al.^140^
miR-31	upregulated	PPP6C, STK40	promotes inflammation and keratinocyte proliferation	Xu et al.^141^
miR-378a-3p	upregulated	BMP2	inhibits keratinocyte proliferation	Soonthornchai et al.^142^
miR-122-5p	upregulated	SPRY2	promotes keratinocyte proliferation	Jiang et al.^143^
miR-126	upregulated	–	promotes keratinocyte proliferation	Feng et al.^144^
miR-150	downregulated	HIF-1α, VEGFA	inhibits keratinocyte proliferation	Li et al.^145^
miR-125b	downregulated	FGFR2, CCR7, and FOXP3	inhibits keratinocyte proliferation	Xu et al.^146^
miR-486-3p	downregulated	TLR4	inhibits keratinocyte proliferation	Jiang et al.^147^

The results of the present study demonstrated that miR-203 expression is significantly upregulated in mice and HaCaT cells stimulated with IL-17. The overexpression of miR-203 inhibits the regulatory effects of p63 and SOCS3, resulting in the continuous activation of STAT3, ultimately leading to excessive proliferation of keratinocytes.^134^ Zhang et al. found that in keratinocytes (KCs), cytokines upregulate the expression of the miR-17-92 cluster by activating the STAT1 signaling pathway. The overexpression of this cluster suppresses cyclin-dependent kinase inhibitor 2B (CDKN2B), thereby promoting keratinocyte proliferation and cell cycle progression.^135^ Wang et al.^136^ found that the overexpression of miR-223 promoted the proliferation of IL-22-stimulated HaCaT cells and reduced apoptosis. The mechanism may be that miR-223 promotes the proliferation of IL-22-stimulated keratinocytes and inhibits their apoptosis through the PTEN/AKT pathway. MiRNAs (such as inhibiting miR-21, miR-31, or miR-210 and increasing miR-125b, etc.) have demonstrated good therapeutic effects in psoriasis,^137^^,^^138^^,^^139^^,^^140^^,^^141^^,^^142^^,^^143^^,^^144^^,^^145^^,^^146^^,^^147^ suggesting that targeting miRNAs is expected to become a new direction for the treatment of psoriasis, but further in-depth research is still needed.

Long non-coding RNAs (lncRNAs) are a class of non-protein-coding RNAs longer than 200 nucleotides that regulate gene expression at multiple levels including epigenetic modification, transcription, post-transcription, translation, and post-translation through structural interactions and/or complementary base pairing with DNA, RNA, or proteins.^148^ LncRNAs play crucial regulatory roles in various skin physiological processes, such as keratinocyte differentiation, melanocyte function, and wound healing.^149^ Multiple studies using microarray or high-throughput RNA sequencing have identified over 4,000 differentially expressed lncRNAs in psoriasis lesional skin, which are associated with keratinocyte differentiation, cell proliferation, apoptosis, and inflammatory immune responses.^150^^,^^151^^,^^152^^,^^153^^,^^154^^,^^155^^,^^156^ Key lncRNAs implicated in keratinocyte proliferation are summarized in Table 3.

**Table 3:** Main long non-coding RNAs altered in psoriasis

name	Level	Target	Function	Reference
MSX2P	upregulated	miR-6731	suppresses keratinocyte proliferation	Qiao et al.^150^
PRINS	downregulated	miR-124, miR-203, miR-21, miR-129, and miR-146a	promotes keratinocyte proliferation	Szegedi et al.^151^
LINC00941	downregulated	SPRR5	suppresses keratinocyte proliferation	Liadaki et al.^152^
MEG3	downregulated	miR-21	suppresses keratinocyte proliferation	Jia et al.^153^
NORAD	upregulated	miR-26a,	promotes keratinocyte proliferation	Li et al.^154^
LOC285194	downregulated	miR-616,	suppresses keratinocyte proliferation	Lin et al.^155^
RP6-65G23.1	upregulated	–	promotes keratinocyte proliferation	Duan et al.^156^

### Metabolic reprogramming regulates keratinocyte proliferation

**Figure 3:**
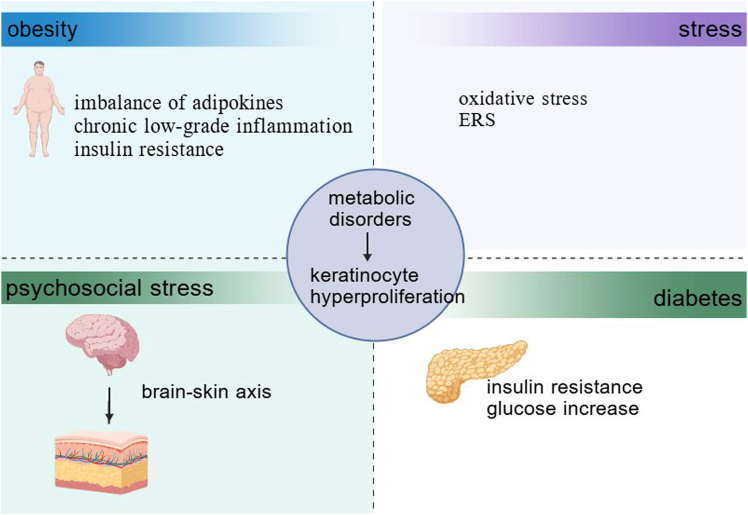
Possible mechanisms linking metabolic disorder and psoriasis The increased release of pro-inflammatory cytokines, adipose tissue secretory adipocytokines, activation of oxidative stress states, increased endoplasmic reticulum stress, insulin resistance, and psychological stress can cause metabolic disorders, thereby inducing the occurrence and development of psoriasis.

Psoriasis also displays altered metabolism (Figure 3).

Obesity is closely associated with the onset and progression of psoriasis.^157^ Through multiple mechanisms, obesity induces systemic dysregulation of lipid metabolism, characterized by elevated circulating triglycerides and free fatty acids (FFAs), as well as reduced high-density lipoprotein cholesterol.^158^ Meanwhile, dysfunctional adipose tissue exhibits increased secretion of pro-inflammatory adipokines (leptin and resistin) and decreased production of anti-inflammatory adipokines (adiponectin), thereby establishing a state of chronic inflammation. Leptin exerts immunomodulatory effects by stimulating macrophages to secrete pro-inflammatory cytokines (TNF-α and IL-6) and may induce the proliferation of Th1 cells.^159^ Furthermore, in psoriasis patients, serum levels of the anti-inflammatory adipokine adiponectin are decreased, which is inversely correlated with the severity of psoriasis manifestations. Elevated FFAs can activate the NLRP3 inflammasome in macrophages, leading to increased secretion of IL-1β and IL-18.^160^ Sphingosine-1-phosphate (S1P), an elevated sphingolipid metabolite in psoriasis patients, acts as both an intracellular second messenger and extracellular G protein-coupled receptor ligand, regulating immune cell trafficking and keratinocyte proliferation/differentiation. Studies have shown that inhibiting S1P lyase to increase S1P levels modulates keratinocyte differentiation, reduces proliferation, and ameliorates IMQ-induced psoriasis-like dermatitis.^161^

Excess lipids accumulate ectopically in non-adipose tissues such as the liver and skeletal muscle, where lipotoxic intermediates (ceramides) induce insulin resistance and cellular dysfunction.^162^^,^^163^ Specifically, sustained FFA elevation, together with increased TNF-α and IL-6, promotes hepatic glucose production and reduces glucose uptake in skeletal muscle. High-glucose and high-fat culture activates the AKT/mTOR pathway, thereby inducing glucose metabolic reprogramming in M5-stimulated keratinocytes.^164^ Glucose is the primary bioenergy source for rapidly proliferating cells and can accelerate glycolysis. Glucose uptake involves glucose transporters, and the expression of GLUT-1 is upregulated in psoriasis.^165^ GLUT-1 is essential for keratinocyte proliferation and stress responses. Specific inhibition of GLUT-1 expression in keratinocytes alleviates epidermal hyperplasia in IMQ-induced psoriasis-like mouse models.^166^ Pyruvate kinase M2 (PKM2) is another key rate-limiting enzyme in glycolysis, with significantly elevated expression levels in psoriasis. PKM2 is critical for keratinocyte proliferation, and conditional knockout of PKM2 in keratinocytes markedly reduces lesion severity in IMQ-induced mouse models.^167^ Recent studies have found that PKM2 regulates NF-κB transcriptional signaling downstream of IL-17 by forming a complex with Act1 and TRAF6, thereby affecting keratinocytes.^168^ CD147/basigin is a molecular marker associated with the hyperproliferation and hypodifferentiation of keratinocytes. CD147 upregulates glucose uptake through interaction with glucose transporters (GLUT1), thereby enhancing glycolytic activity and exacerbating psoriasis inflammation.^169^ Liu et al. demonstrated that basal epidermal cells compete for glucose in psoriasis by promoting oxidative phosphorylation and upregulating COX7B via glucose metabolism and ROS production, which maintains keratinocyte hyperproliferation.^170^ In patients with psoriasis lesions, macrophages, serum CD4^+^ T cells, and neutrophils with high CXCR4 expression all exhibit increased glycolytic levels.^171^ These cells undergo metabolic shifts toward glycolysis and display enhanced pro-inflammatory functions.^172^^,^^173^

In terms of oxidative stress, UV radiation, environmental pollution, and other factors induce excessive production of reactive oxygen species (ROS) in keratinocytes. ROS can oxidatively damage mitochondrial DNA, inhibit mitochondrial respiratory chain function, and reduce ATP production via aerobic respiration, forcing keratinocytes to switch to glycolytic metabolism to maintain energy supply. Meanwhile, ROS activates the NF-κB signaling pathway, upregulating the expression of GLUT1 and glycolytic enzymes to further enhance glycolytic activity.^174^ Additionally, ROS oxidize intracellular lipids to generate lipid peroxides (malondialdehyde), which can promote the expression of keratinocyte proliferation-related genes by activating the MAPK signaling pathway.^175^^,^^176^ Simultaneously, ROS-induced accumulation of low-density lipoprotein (LDL) enhances lipid peroxidation, which activates cyclic guanosine monophosphate (cGMP) and decreases cyclic adenosine monophosphate (cAMP) levels, leading to the exacerbated epidermal proliferation observed in psoriasis patients.^177^ Endoplasmic reticulum (ER) stress is present in patients with psoriasis. The ER stress sensor inositol-requiring enzyme 1 can contribute to obesity by inducing dysfunction in both brown and white adipose tissues. Furthermore, reducing ER stress has been demonstrated to ameliorate insulin resistance.^178^

The specific mechanisms underlying stress-induced psoriasis remain incompletely understood. Although existing research findings are inconsistent, substantial evidence indicates differences in physiological stress responses between psoriasis patients and healthy individuals, characterized by alterations in the HPA axis and sympathetic-adrenal-medullary (SAM) system function.^179^^,^^180^ Psychological stress may induce leukocyte redistribution, promoting the recruitment of inflammatory cells to the skin.^181^ Furthermore, interactions between neuro-related factors (e.g., vasoactive intestinal peptide) released by peripheral sensory nerves and mast cells may contribute to pathogenesis by promoting neurogenic inflammation.^182^ Moreover, psoriasis lesions exhibit abnormal upregulation of lipid mediators such as lysophosphatidylcholine (LPC). Acting as a non-H^+^ ligand for acid-sensing ion channel 3 (ASIC3), LPC activates sensory neurons to release calcitonin gene-related peptide (CGRP). Through neuro-immune-metabolic crosstalk, this process further induces keratinocyte proliferation and IL-23 secretion, exacerbating lesion progression.^183^

In addition to the circulation, amino acid levels in the skin also differ between psoriasis patients and healthy individuals. In psoriasis, aberrant activation of glutaminase 1 (GLS1) mediates excessive glutamine consumption. IL-17A enhances GLS1 expression in keratinocytes via the MALT1/c-Jun pathway, leading to hyperproliferation of keratinocytes and increased chemokine production. Inhibition of either GLS1 or the MALT1 protease suppresses Th17 and γδ T17 cell differentiation and ameliorates psoriasis-like epidermal hyperplasia in mouse models.^184^

### Autophagy dysfunction and keratinocyte proliferation

Olufolake et al. compared the skin of psoriasis patients with that of healthy adults and found that several autophagy markers, like LC3, were not expressed in psoriasis lesions. In contrast, these autophagy markers were normally expressed in healthy adults.^185^ In addition, TNF-α, a key proinflammatory cytokine in psoriasis, inhibits autophagy and lysosomal function of keratinocytes,^186^ indicating a dysfunction of autophagy in psoriasis. The inactivation of MAPK also contributes to the decreased keratinocyte autophagy, leading to the development of psoriasis.^187^

Protein p62, also known as SQSTM1, links the ubiquitination and autophagy machineries. Defective autophagy enhanced the activity of p62, which plays key roles in keratinocyte proliferation and cell cycle progression.^188^ PIN1 is a unique enzyme that isomerizes the target protein proline residues. PIN1 silencing protects keratinocyte from M5-induced hyperproliferation and inflammatory damage in psoriasis by activating mitochondrial autophagy.^189^ Lee discovered that impaired autophagy in myeloid cells promoted neutrophil recruitment and aggravated psoriasis skin inflammation through a significant effect on the IL-1β-IL1R1/CXCL2 axis. The hyperactivated inflammatory response promotes keratinocyte proliferation in psoriasis.^190^ High-mobility group box 1 (HMGB1) is a nuclear protein that is released by activated macrophages. HMGB1 contributes to inflammation by inducing proinflammatory responses through Toll-like receptor 4 (TLR4). Autophagy-based unconventional secretion of HMGB1 by keratinocytes plays a pivotal role in psoriasis skin inflammation and keratinocyte proliferation. rHMGB1 significantly induced psoriasis-associated inflammatory medium expression and reduced the proliferation of keratinocytes.^191^ ULK1, the key autophagic initiator, and its phosphorylation at Ser556 were distinctively decreased in the epidermis from lesional skin of psoriasis patients; selective ULK1 inhibitor significantly arrested keratinocyte proliferation and promoted apoptosis of keratinocytes.^192^

### Keratinocyte proliferation and microbiome

Recent studies have revealed a close association between the skin microbiota and psoriasis. Fry and Baker’s research indicates that certain bacteria (*Staphylococcus aureus* and *Streptococcus pyogenes*), viruses (human papillomavirus [HPV] and endogenous retrovirus), and fungi (Malasella, *Candida albicans*) may all aggravate psoriasis.^193^

Among these, *S. aureus* and *Streptococcus pyogenes* may play significant roles in psoriasis. *S. aureus* can stimulate the production of antimicrobial peptides (AMPs), IL-12, and IL-23, promoting Th17 cell differentiation.^194^ This leads to the production of pro-inflammatory mediators such as IL-17A, TNF-α, IFN-α, and IL-22, resulting in excessive proliferation of keratinocytes and infiltration of immune cells into the skin, ultimately exacerbating psoriasis inflammation.

Streptococcus, particularly beta-hemolytic Streptococcus, is abundant in psoriasis skin and serves as a clinically significant trigger for chronic plaque psoriasis.^195^ This bacterium carries streptococcal M protein and streptococcal peptidoglycan, which function as superantigens (SAgs) to activate T lymphocytes. Streptococcal peptidoglycans bind to MHC class II molecules and T cell receptors, initiating specific pathological responses and releasing cytokines.^196^ Malassezia exacerbates psoriasis by upregulating the expression of TGF-β, heat shock protein 70 (HSP70), and integrin chains in human keratinocytes, leading to epidermal hyperproliferation and cell migration.^197^The αβ T cells sensitized by *Candida albicans* can secrete IL-17, promoting the persistence of the disease and the acute attack of psoriasis.^198^ Besides, human immunodeficiency virus (HIV) and HPV infections are associated with more severe psoriasis phenotypes.^199^

### Other factors that affect keratinocyte proliferation

Numerous factors influence keratinocyte proliferation. In addition to those mentioned above, several others are listed below.

Emerging evidence has uncovered the active involvement of the sensory nervous system in psoriasis pathogenesis. Sensory neurons can directly participate in the excessive proliferation of keratinocytes during the formation of psoriasis lesions without relying on the immune system by releasing CGRP, a neuropeptide released by sensory neurons.^200^ Notably, central or peripheral nerve injury alleviates psoriasis, and epidural injection of lidocaine, a nonselective voltage-gated sodium channel blocker, has been shown to markedly reduce keratinocyte proliferation in both patients and imiquimod-induced mouse models.^201^^,^^202^ Qiao et al. demonstrated that mechanical stretch can exacerbate psoriasis lesions by promoting cell proliferation and amplifying the production of proinflammatory cytokines by keratinocytes.^203^ Nicotine can promote the secretion of cytokines such as IL-12, IL-2, TNF, INF-α, and granulocyte-macrophage colony-stimulating factor through the activation pathway, thereby promoting the proliferation of keratinocytes.^204^ Studies have shown that alcohol and acetone can stimulate the proliferation of keratinocytes and increase the mRNA levels of characteristic genes of keratinocytes in the proliferative phase (α5 integrin, cyclin D1, and keratinocyte growth factor receptor).^205^ Obesity is known as a low-grade chronic inflammatory process with elevated levels of C-reactive protein, IL-6, TNF, and leptin. Leptin exerts immunomodulatory effects by stimulating macrophages to produce pro-inflammatory cytokines (such as TNF-α and IL-6) and may induce the proliferation of Th1 cells, leading to rapid proliferation of skin cells.^206^^,^^207^

## Conclusion

**Figure 4:**
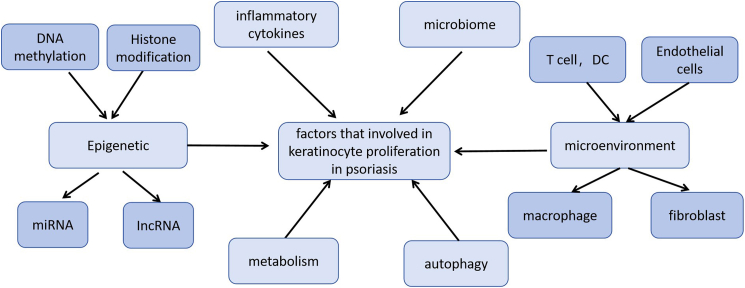
Factors influencing the proliferation of keratinocytes Factors including inflammatory cytokines, epigenetics, metabolism, autophagy, microbiome, and the immune microenvironment influence the proliferation of keratinocytes.

Psoriasis is an immune-mediated disease driven by a combination of genetic and environmental factors and characterized primarily by the abnormal proliferation of keratinocytes. Inflammatory cytokines, microRNAs, epigenetics, metabolism, autophagy, microbiome, and the immune microenvironment influence keratinocyte proliferation (Figure 4).

With the emergence of new multi-omics tools such as single-cell metabolomics and spatial metabolomics, it is possible and necessary to construct a multi-dimensional metabolic network of psoriasis. We believe that establishing a psoriasis network centered on metabolism will be the hot topic in the next decade. Besides, targeting specific microbial and epigenetic markers and defining associated molecular signatures will pave the way for novel psoriasis therapeutics.

## Acknowledgments

The authors thank the funding support by the National Natural Science Foundation of Republic of China (NFSC #82273539) and the Nature Science Foundation of Shanxi Province (#202203021222411 and # 202203021212019).

## Declaration of interests

The authors declare no conflict of interest.
